# The AQUA-FONTIS study: protocol of a multidisciplinary, cross-sectional and prospective longitudinal study for developing standardized diagnostics and classification of non-thyroidal illness syndrome

**DOI:** 10.1186/1472-6823-8-13

**Published:** 2008-10-13

**Authors:** Johannes W Dietrich, Axel Stachon, Biljana Antic, Harald H Klein, Steffen Hering

**Affiliations:** 1 Medical Hospital 1, Bergmannsheil University Hospitals, Ruhr University of Bochum, Bochum, NRW, Germany; 2 Institute of Clinical Chemistry, Transfusion and Laboratory Medicine, Bergmannsheil University Hospitals, Ruhr University of Bochum, Bochum, NRW, Germany; 3 Klinik für Innere Medizin, Evangelisches Krankenhaus Hattingen, Hattingen, NRW, Germany

## Abstract

**Background:**

Non-thyroidal illness syndrome (NTIS) is a characteristic functional constellation of thyrotropic feedback control that frequently occurs in critically ill patients. Although this condition is associated with significantly increased morbidity and mortality, there is still controversy on whether NTIS is caused by artefacts, is a form of beneficial adaptation, or is a disorder requiring treatment. Trials investigating substitution therapy of NTIS revealed contradictory results. The comparison of heterogeneous patient cohorts may be the cause for those inconsistencies.

**Objectives:**

Primary objective of this study is the identification and differentiation of different functional states of thyrotropic feedback control in order to define relevant evaluation criteria for the prognosis of affected patients. Furthermore, we intend to assess the significance of an innovative physiological index approach (SPINA) in differential diagnosis between NTIS and latent (so-called "sub-clinical") thyrotoxicosis.

Secondary objective is observation of variables that quantify distinct components of NTIS in the context of independent predictors of evolution, survival or pathophysiological condition and influencing or disturbing factors like medication.

**Design:**

The **a**pproach to a **qua**ntitative **f**ollow-up **o**f **n**on-**t**hyroidal **i**llness **s**yndrome (AQUA FONTIS study) is designed as both a cross-sectional and prospective longitudinal observation trial in critically ill patients. Patients are observed in at least two evaluation points with consecutive assessments of thyroid status, physiological and clinical data in additional weekly observations up to discharge. A second part of the study investigates the neuropsychological impact of NTIS and medium-term outcomes.

The study design incorporates a two-module structure that covers a reduced protocol in form of an observation trial before patients give informed consent. Additional investigations are performed if and after patients agree in participation.

**Trial Registration:**

ClinicalTrials.gov NCT00591032

## Background

Non-thyroidal illness syndrome (NTIS or euthyroid sick syndrome) is a complex endocrine condition that may occur in critically ill patients. It is associated with significant deterioration of prognosis.

NTIS is characterised by three components that may occur single or in combination:central hypothyroidism (transient thyrotropic insufficiency), impaired protein binding of thyroid hormones and reduced formation of T3 with simultaneously increased conversion to rT3 (low-T3-syndrome) [1,2].

In 1973, characteristic alterations of thyroid metabolism have been first described in the starving organism [3,4]. Additional observations could reveal that these alterations are also common in critically ill patients where they form the correlate of endocrine dysregulation with increased morbidity and mortality [5-8].

However, explanation concepts for this complex constellation are different. Up to now, in essence five hypotheses are discussed in literature [2]:

1. All observed abnormalities are the result of test artefacts. In reality, the patients are euthyroid.

2. The changes in the levels of peripheral thyroid hormones mirror the effect of certain binding inhibitors that influence

A) laboratory results only or

B) also the transfer of thyroid hormones into the tissue of diseased persons and thus diminish binding of iodothyronines to T3 receptors.

3. Due to increased local deiodation, T3-levels are normal in the pituitary gland while they are low in the rest of the organism.

4. Levels of peripheral thyroid hormones are actually low so that affected patients are biochemically hypothyroid. However, this useful physiological function should not be manipulated.

5. NTIS is secondary or tertiary hypothyroidism. The resulting tissue hypothyroidism should be treated with appropriate substitution therapy [2].

Despite of intensive and long lasting research to many of its details NTIS is still poorly characterized in an integrative view. Additionally, a clinically usable classification is lacking.

Given the fact that patients with NTIS are faced with poor prognosis, several studies have been conducted in the past evaluating the question of possible treatment [2,7]. However, they did not yield unambiguous results. Some studies could show a benefit of substitution therapy with thyroid hormones, e.g. regarding the incidence of atrial fibrillation [2,7,9,10] and hemodynamic parameters [11,12] while others could not observe relevant differences in outcome between treated and untreated patients [13-15]. Several studies even described detrimental effects of substitution therapy ranging from increased risk of hyperthyroidism [16,17] over undesirably high protein catabolism [18] to severe side effects in patients with adrenal insufficiency that may be difficult to identify during critical illness [19]. Recently, a small trial investigating the effect of selenium substitution on the development of critically ill patients demonstrated improvements in prognosis, but, in spite of the known selenium-dependency of peripheral deiodinases, this outcome was not caused by a direct effect on thyroid homeostasis [20].

The problem of inconclusive and partly contradictory study results is aggravated by the fact that there is no consistent definition of NTIS that delimits this constellation from euthyroid state and that weighs the associated components regarding their relevance. Therefore, it may be assumed that the mentioned studies compared inhomogeneous patient groups.

An additional challenge affecting clinical practice is the fact that partial thyrotropic insufficiency in the course of NTIS can hardly be distinguished from latent ("subclinical") hyperthyroidism – although pathophysiology and therapeutic implications are opposed.

### Objectives of the AQUA FONTIS project

The AQUA FONTIS study (**a**pproach to a **qua**ntitative **f**ollow-up **o**f **n**on-**t**hyroidal **i**llness **s**yndrome) is primarily intended to develop a clear-cut definition and classification of NTIS (Table 1). Overall, this project is proposed to deliver a prognostic aid by providing a differentiated classification, to contribute to a standardised, rational and inexpensive diagnostic procedure in form of quantitative indices, and to lay the foundation for future therapeutic trials by identifying subgroups that may benefit from therapy.

**Table 1:** Proposed "HPD" classification

**Facet**	**Possible values**
Hypothalamic-pituitary dysfunction or adaptation ("H")	H0: Normal TSH secretion, adequate to T4 level H1: Thyrotropic insufficiency
	
Impaired plasma protein binding ("P")	P0: Normal binding to plasma proteins P1: Normal binding with reduced levels of plasma proteins P2: reduced binding to proteins within normal levels P3: reduced binding and reduced protein levels
	
Reduced deiodation ("D")	D0: Normal deiodation D1: Reduced deiodation activity

Example: H1P0D1: Thyrotropic insufficiency with reduced deiodation and normal protein binding.

Outcome measure of this primary objective is the comparison of different decision criteria as presented in Table 2. These criteria are assessed with regard to the prognosis of the patients. Additionally, the significance of an innovative physiological index approach (SPINA) [21] is to be evaluated regarding its applicability for differential diagnosis between NTIS and latent thyrotoxicosis. It will therefore be evaluated in terms of sensitivity, specificity and likelihood ratios with ROC analysis.

**Table 2:** Decision criteria

**Criterion**	**Definition**
Thyrotropic insufficiency	a) Reduced levels of both TSH and free T4. b) Thyrotroph T4 Sensitivity Index (TTSI) reduced below its reference interval.
	
Reduced plasma protein binding	a) Reduced relative ratios of bound and free hormone levels b) Reduced apparent dissociation constant of T3 at thyroxin binding globulin (K_30_)
	
Reduced deiodation	a) Reduced level for free T3 b) diminished T3/T4-ratio c) reduced sum activity of peripheral 5'-Dejodinase (G_D_^)

As secondary objective, we plan to observe variables that quantify distinct components of NTIS in the context of independent predictors of evolution, survival or pathophysiological condition as well as influencing or disturbing factors like medication or medical procedures. Outcome measures are correlations between quantitatively described components and external factors as well as their distributions in the context of dichotomic influencing factors.

## Methods

**Figure 1:**
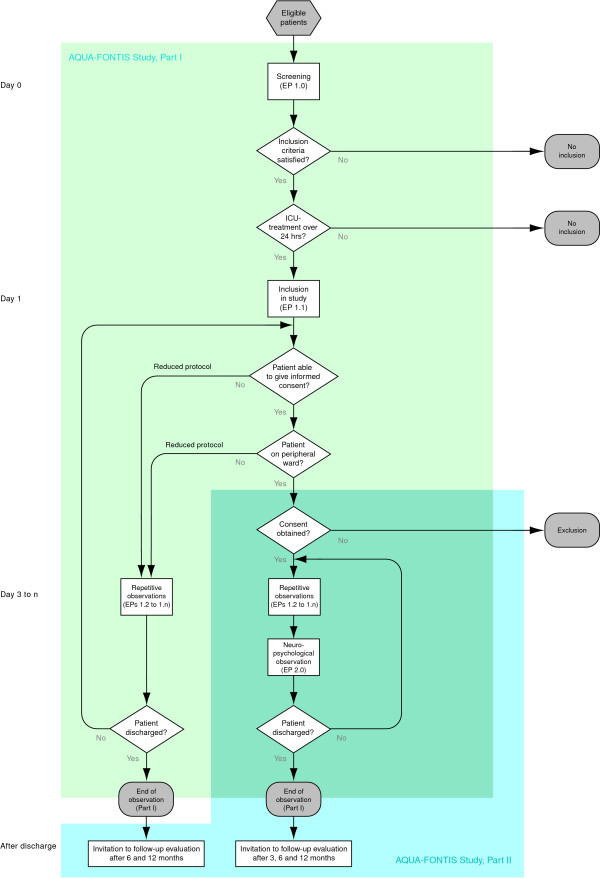
**Flowchart of the AQUA-FONTIS study with its two intertwined modules.** See text and table 5 for detailed information.

The AQUA FONTIS study is designed as both a cross-sectional and prospective longitudinal observation trial (Figure 1).

### Recruitment and criteria for inclusion and exclusion

This study recruits critically ill patients treated in one medical and two surgical intensive care units of the Bergmannsheil University hospitals for evaluation of integrative thyrotropic control and follow-up. Inclusion and exclusion criteria are presented in table 3. The diagnostic criteria are based on the ICD-10 system [22].

**Table 3:** Inclusion and exclusion criteria of the AQUA FONTIS study

**Inclusion**	**Exclusion**
Severe illness requiring intensive care	Substituted hypothyroidism (E03.0, E03.1, E03.3 E03.9, E89.0) or substitution in case of thyroid carcinoma (C73)
	
Stay of at least 24 hours at the ICU	Hyperthyroidism treated with thyrostatic agents and exhibiting a TSH level not below the reference region (E05.0 E05.9, E06.2)
	
	Manifest AIDS disease (B24)
	
	Pregnancy

### Decision criteria

Latent (so called "subclinical") thyrotoxicosis is defined as suppressed thyrotropin levels with free T4 levels being in their reference intervals and additional clinical evidence for tissue hyperthyroidism (suggestive history or Burch-Wartofsky point scale of more than 45 points [23]). Decision criteria to be evaluated are reproduced in Table 2.

### Calculations

Thyrotroph T4 Sensitivity Index (TTSI) is calculated as

(1) $$TTSI=\frac{100[TSH][FT_{4}]}{l_{u}}$$

with *l*_*u *_being the upper limit of the reference interval for free T4 [24]. Theoretical secretion capacity of the thyroid gland (G_T_^) is computed with

(2) $${\hat{G}}_{T}=\frac{\beta_{T}(D_{T}+[TSH])(1+K_{41}[TBG]+K_{42}[TBPA])[FT_{4}]}{\alpha_{T}[TSH]},$$

where parameters are taken from Table 4[21].

**Table 4:** Parameters for structure parameter inference [21]

**Symbol**	**Explanation**	**Value**
α_T_	Dilution factor for T4 (reciprocal of apparent volume of distribution)	0,1 l^-1^
β_T_	Clearance exponent for T4	1,1 * 10^-6 ^sec^-1^
D_T_	EC_50 _for TSH	2,75 mU/l
K_41_	Dissociation constant T4-TBG	2 * 10^10 ^l/mol
		
K_42_	Dissociation constant T4-TBPA	2 * 10^8 ^l/mol
		
α _31_	Dilution factor for T3	0,026 l^-1^
β _31_	Clearance exponent for T3	8 * 10^-6 ^sec^-1^
K_M1_	Dissociation constant of type-1-deiodinase	5 * 10^-7 ^mol/l
		
K_30_	Dissociation constant T3-TBG	2 * 10^9 ^l/mol

Sum activity of peripheral 5'-deiodinase (G_D_^) is defined with

(3) $${\hat{G}}_{D}=\frac{\beta_{31}(K_{M1}+[FT_{4}])(1+K_{30}[TBG])[FT_{3}]}{\alpha_{31}[FT_{4}]},$$

parameters are again from Table 4[21].

Apparent dissociation constant of T3 binding to TBG is obtained with

(4) $${\hat{K}}_{30}=\frac{\left[T_{3}]-[FT_{3}\right]}{\left[TBG][FT_{3}\right]}$$

from levels of iodothyronines and TBG.

Relative binding ratio is calculated with

(5) $$R_{B}=\frac{\frac{\left[T_{3}\right]}{l_{l,TT3}}}{\frac{\left[FT_{3}\right]}{l_{l,FT3}}}$$

where *l*_*l*, *TT*3 _denotes the lower limit of the reference interval for TT3 and *l*_*l*, *FT*3 _is the lower limit of the reference interval for FT3.

### Follow-up and Evaluation Points

Patients are subjected to at least one evaluation point. Critically ill persons that are newly admitted to an ICU are screened in the first EP (1.0) for fulfilment of the inclusion and exclusion criteria from Table 3 and entered in the database (see below). The second EP (1.1) is performed after 24 hours; only those eligible patients that are still under intensive care will be included in the study and subsequently prospectively observed. On the occasion of this and the subsequent EPs (after 72 hours for EP 1.2 and weekly in the further course of the study), severity scores like SAPS II and APACHE II as well as physiological parameters and data regarding pharmacological therapy or medical interventions are surveyed (Table 5). Additionally, laboratory investigations that are part of routine diagnostics (e.g. TSH, FT4, total T3, inflammation parameters, total protein etc.) are complemented by those parameters that are collected for the study (total T4, free T3 and antibodies against iodothyronines as well as thyroid tissue). As all required specimens are taken in the course of routine diagnostic procedures, additional assays, but no additional blood samples are necessary.

**Table 5:** Schedule of evaluation points

	EP 1.0	EP 1.1	EP 1.2	EP 1.3	EP 1.999
	Screening	After 24 hours	After 72 hours	Weekly	After discharge

**Screening**					
Inclusion and Exclusion criteria	•	•			
Informed consent			°	°	°
**Demography**					
Age, Gender					•
History	•				
**Laboratory examinations**					
TSH, TT4, FT4, TT3, FT3		•	•	•	
rT3			•		
Total protein, Albumin		•	•	•	
BC, CRP, ATIII, Fibrinogen		•	•	•	
Thyroid antibodies, T4/T3-Abs.		•			
**Other investigations**		•	•	•	
Temperature, Horowitz-Quotient					
Vital signs	•	•	•	•	
**Evolution data**					
Medication	•	•	•	•	•
APACHE-II-/SAPS-II-Score, GCS		•	•	•	•
Diagnosis		•			•
HD/CVVH/CVVHDF	•	•	•	•	•
IABP	•	•	•	•	•
Persistent organ failure					•
Outcome (survival etc.)					•

	EP 2.0	EP 2.1	EP 2.2	EP 2.3	

	After transfer to peripheral ward	After 3 months	After 6 months	After 1 year	

**History and clinical data**					
Burch-Wartofsky score	°	°			
Medication	°	°	°	°	
Vital signs	°	°	°	°	
**Laboratory examinations**					
TSH, TT4, FT4, TT3, FT3	°	°			
**Neuropsychological data**					
Number connection test	°	°			
RWT	°	°			
SKT	°	°			
HADS	°				
SF36		°	°	°	
**Other investigations**					
Thyroid ultrasonography	°	°			
Outcome (survival etc.)	°	°	°	°	

•: Scheduled at indicated EP.

°: Scheduled after transfer to peripheral ward.

When patients have been transferred to a peripheral ward they are subjected to a single additional evaluation point (EP 2.0) surveying neuropsychological data covering anxiety, depression, speed of information processing and memory (see second part of Table 5). After discharge, they will be invited to repeated neuropsychological evaluations after 3, 6 and 12 months. This second part of the study is restricted to patients that gave informed consent (see below) and that have the mental and somatic ability to take part in this sub-study.

Patients that are admitted to an ICU a second time will only be observed up to that date, i.e. follow-up observation will stop after readmission to the ICU.

### Sample size and power calculation

The AQUA-FONTIS study has a planned sample size of 650 patients. On the premise of a beta level of 0.1 (corresponding to a power of 0.9), an alpha level of 0.05, and standard deviations and event rates as observed in the literature and in this study to date, between 200 and 566 patients would be required, depending on the objective in question.

With an appropriate safety-margin a total required number of 650 patients results. Most questions could be answered with 500 patients, however.

### Data management

Data are stored as electronic case report forms (eCRF) on a Macintosh server in a network-based relational database that has been developed with FileMaker Pro 5.5 (FileMaker, Inc., Santa Clara, CA, USA). It accommodates important principles of data protection by the fact that patient data are stored in pseudonymised form, i.e. they are referred to by number only, while mapping to patient names is only possible with an external list that is stored separately from the database. Additionally, the server is operating in the hospital's intranet with access from outside being blocked by a firewall. Data security is provided by a combination of established methods that cover regular backups and archiving as well as galvanic isolation of the server by an uninterruptible power supply. Operation of the server in a locked, dedicated room that is inaccessible for unauthorized persons serves both data protection and data security.

Other benefits of this database-founded approach are the ability to store very large amounts of information and the option to enter data from several wards simultaneously.

### Statistics

The main null-hypothesis to be tested is that the decision-criteria presented in Table 2 lead to results that are independently distributed from the prognosis of included patients. Risk stratification will be performed with log rank test and Cox regression; end points are mortality, length of stay in hospital (LOSIH), length of stay in intensive care unit (LOSICU) and ability to work after discharge.

Cross sectional analysis will be done with the chi-squared test (for categorial data) and Student's t-test (for continuous data).

Multivariate regression analysis will be used to investigate correlation between quantitatively determined components of thyroid homeostasis and influencing pathophysiological factors.

Missing data analysis will be performed with "modern" procedures like EM algorithm and multiple imputation [25], if required.

### Ethical considerations

For various reasons, most critically ill patients are not able to give informed consent [26,27]. We therefore restricted investigations in the first part of the trial to observation and laboratory tests from sera that have been obtained for routine diagnostics independently from this study, therefore not requiring extra-specimens. In essence, the reduced sub-protocol is designed in form of an observational study that resembles epidemiological investigations; this part of the study is therefore not subject of the German law governing clinical trials of drugs and medicinal products (Arzneimittelgesetz) and the corresponding European directives 2001/20/EC and 2005/28/EC [28-30].

After being transferred to a peripheral ward, each participant receives information about the trial both verbally and in written form. Continued participation is voluntary, without expected negative side effects, and the patient can withdraw his or her consent at any time for parts of the study (e.g. future evaluation points) or the whole trial without consequence for treatment possibilities. All patients will receive a copy of their rights.

Patients that are never able to give consent, e.g. because they die before regaining consciousness, or that acquire permanent brain damage, only traverse the simplified procedure of the reduced observational protocol as described above. The same holds true for patients that have been discharged or transferred to an external hospital before being asked for consent, or that are not able to communicate in German.

The local ethics committee of the Ruhr-University of Bochum has approved the protocol under the file number 2848. The trial is registered at ClinicalTrials.gov as NCT00591032.

Both significant and insignificant findings from the trial will be published, in accordance with the STROBE statement [31]. Links to publications will be made available on the study-website .

### Status of the study

Up to now, 561 patients have been screened, 470 persons are included in the study, corresponding to 72% of the planned sample size.

In this unselected sample, currently 210 females (37%) have been observed, the ages of included patients ranging between 9 and 96 years. 61% of patients were screened from the two surgical ICUs, 39% from the medical ICU.

## Discussion

The **a**pproach to a **qua**ntitative **f**ollow-up **o**f **n**on-**t**hyroidal **i**llness **s**yndrome (AQUA-FONTIS study) has been designed to develop standardised diagnostic and classification criteria for NTIS. Apart from identification and differentiation of distinct functional states of thyrotropic feedback control in the context of critical illness, this study will help to assess the correlation of variables that quantify the components of NTIS with independent predictors of evolution, survival or pathophysiological condition. Additionally, it will evaluate the effect of influencing or disturbing factors like medication or interventional procedures.

In the past, many studies dealt with the impact of severe diseases on thyroid metabolism [1-8]. However, they had inconsistent results, which may be the result of lacking standardised criteria. The objective of the AQUA FONTIS project is to provide more clear-cut decision criteria for prognosis and possibly therapy of affected patients. These may help to make future studies comparable.

However, also the AQUA FONTIS study is faced with several limitations. These predominantly accrue from the fact that most patients receiving intensive care are not able to give informed consent [26,32]. We therefore had to constrain the possible investigations to methods that are purely observational or use patient sera that were obtained for routine diagnostics. Due to their extensive ethical and legal implications, we were e.g. not able to investigate genetic aspects of NTIS without informed consent: Although deferred consent for genetic investigations could be obtained from surviving patients this is by nature not possible in persons that are admitted as emergency cases and die early, so that a relevant bias would be to expect [27].

Additionally, the fact that a high proportion of patients will be admitted in cases of accidents or emergency makes it difficult to control relevant influencing factors. Conditions like existing thyroid diseases or previous medication may therefore in some cases be missed. Furthermore, stratification will be impossible.

These restrictions are shared by all observational studies. Alternative forms of data acquisition may seem more appropriate from a pure statistical point of view. However, the mentioned medical and ethical marginal conditions render them impossible.

Its two-module structure, covering an observational part before patients gave consent and a more comprehensive sub-study that is traversed after consent was obtained, helps to overcome some of the limitations of the AQUA FONTS study. Additionally, the number of patients that could already be included up to now gives reason to believe that some of the poorly controllable influencing factors may partly be compensated by a large final case number. We are therefore convinced that this study will help to identify decision-parameters for defining states of particular importance with respect to the prognosis of affected patients. These may lay the basis for future epidemiological or therapeutic trials.

## Abbreviations

APACHE: acute physiology and chronic health evaluation; AQUA FONTIS: approach to a quantitative follow-up of non-thyroidal illness syndrome; BWPS: Burch-Wartofsky point scale; eCRF: electronic case report form; EP: evaluation point; GCS: Glasgow come scale; HADS: hospital anxiety and depression scale; ICU: intensive care unit; LOSICU: length of stay in intensive care unit; LOSIH: length of stay in hospital; NTIS: non-thyroidal illness syndrome; rT3: reverse triiodothyronine; RWT: Regensburger Wortflüssigkeitstest (Regensburg word fluidity test); SF36: short form with 36 items; SKT: Syndrom-Kurztest (syndrome short evaluation); SPINA: structure parameter inference approach; T3: triiodothyronine; T4: levothyroxine; TSH: thyrotropin; TTSI: thyrotroph T4 sensitivity index.

## Competing interests

The authors declare that they have no competing interests.

## Authors' contributions

All authors were involved in conception and design of the study protocol, drafting or revising the manuscript, and have approved the final manuscript. JWD is responsible for performing evaluation points, data analysis and overall coordination. AS is responsible for laboratory assays, SH and HHK are co-responsible for data analysis. BA was in the planning phase of the study responsible for the development of the ethical conception. All authors will participate in interpretation of results.

## Pre-publication history

The pre-publication history for this paper can be accessed here:



## References

[B1] Berghe G Van den (2002). Dynamic neuroendocrine responses to critical illness. Front Neuroendocrinol.

[B2] De Groot LJ (2006). Non-thyroidal illness syndrome is a manifestation of hypothalamic-pituitary dysfunction, and in view of current evidence, should be treated with appropriate replacement therapies. Crit Care Clin.

[B3] Rothenbuchner G, Loos U, Kiessling WR, Birk J, Pfeiffer EF (1973). The influence of total starvation on the pituitary-thyroid-axis in obese individuals. Acta Endocrinol Suppl (Copenh).

[B4] Portnay GI, O'Brian JT, Bush J, Vagenakis AG, Azizi F, Arky RA, Ingbar SH, Braverman LE (1974). The effect of starvation on the concentration and binding of thyroxine and triiodothyronine in serum and on the response to TRH. J Clin Endocrinol Metab.

[B5] Chopra IJ (1996). Nonthyroidal illness syndrome or euthyroid sick syndrome?. Endocr Pract.

[B6] Iervasi G, Pingitore A, Landi P, Raciti M, Ripoli A, Scarlattini M, L'Abbate A, Donato L (2003). Low-T3 syndrome: a strong prognostic predictor of death in patients with heart disease. Circulation.

[B7] De Groot LJ (1999). Dangerous dogmas in medicine: the nonthyroidal illness syndrome. J Clin Endocrinol Metab.

[B8] Scoscia E, Baglioni S, Eslami A, Iervasi G, Monti S, Todisco T (2004). Low triiodothyronine (T3) state: a predictor of outcome in respiratory failure? Results of a clinical pilot study. Eur J Endocrinol.

[B9] Klemperer JD, Klein IL, Ojamaa K, Helm RE, Gomez M, Isom OW, Krieger KH (1996). Triiodothyronine therapy lowers the incidence of atrial fibrillation after cardiac operations. Ann Thorac Surg.

[B10] Klemperer JD, Klein I, Gomez M, Helm RE, Ojamaa K, Thomas SJ, Isom OW, Krieger K (1995). Thyroid hormone treatment after coronary-artery bypass surgery. N Engl J Med.

[B11] Ranasinghe AM, Quinn DW, Pagano D, Edwards N, Faroqui M, Graham TR, Keogh BE, Mascaro J, Riddington DW, Rooney SJ, Townend JN, Wilson IC, Bonser RS (2006). Glucose-insulin-potassium and tri-iodothyronine individually improve hemodynamic performance and are associated with reduced troponin I release after on-pump coronary artery bypass grafting. Circulation.

[B12] Wyne KL (2005). The role of thyroid hormone therapy in acutely ill cardiac patients. Crit Care.

[B13] Magalhaes AP, Gus M, Silva LB, Schaan BD (2006). Oral triiodothyronine for the prevention of thyroid hormone reduction in adult valvular cardiac surgery. Braz J Med Biol Res.

[B14] Guden M, Akpinar B, Saggbas E, Sanisoglu I, Cakali E, Bayindir O (2002). Effects of intravenous triiodothyronine during coronary artery bypass surgery. Asian Cardiovasc Thorac Ann.

[B15] Ronald A, Dunning J (2006). Does perioperative thyroxine have a role during adult cardiac surgery?. Interact Cardiovasc Thorac Surg.

[B16] Acker CG, Singh AR, Flick RP, Bernardini J, Greenberg A, Johnson JP (2000). A trial of thyroxine in acute renal failure. Kidney Int.

[B17] Brent GA, Hershman JM (1986). Thyroxine therapy in patients with severe nonthyroidal illnesses and low serum thyroxine concentration. J Clin Endocrinol Metab.

[B18] Utiger RD (1995). Altered thyroid function in nonthyroidal illness and surgery. To treat or not to treat?. N Engl J Med.

[B19] Caplan RH (1999). Comment on dangerous dogmas in medicine: the nonthyroidal illness syndrome. J Clin Endocrinol Metab.

[B20] Angstwurm MW, Schopohl J, Gaertner R (2004). Selenium substitution has no direct effect on thyroid hormone metabolism in critically ill patients. Eur J Endocrinol.

[B21] Dietrich JW, Fischer MR, Jauch J, Pantke E, Gärtner R, Pickardt CR (1999). SPINA-THYR: A novel systems theoretic approach to determine the secretion capacity of the thyroid gland. European Journal of Internal Medicine.

[B22] Anonymous International Classification of Diseases (ICD).

[B23] Burch HB, Wartofsky L (1993). Life-threatening thyrotoxicosis. Thyroid storm. Endocrinol Metab Clin North Am.

[B24] Pohlenz J, Weiss RE, Macchia PE, Pannain S, Lau IT, Ho H, Refetoff S (1999). Five new families with resistance to thyroid hormone not caused by mutations in the thyroid hormone receptor beta gene. J Clin Endocrinol Metab.

[B25] Graham JW (2008). Missing Data Analysis: Making It Work in the Real World. Annu Rev Psychol.

[B26] Druml C (2004). Informed consent of incapable (ICU) patients in Europe: existing laws and the EU Directive. Curr Opin Crit Care.

[B27] Jansen TC, Kompanje EJ, Druml C, Menon DK, Wiedermann CJ, Bakker J (2007). Deferred consent in emergency intensive care research: what if the patient dies early? Use the data or not?. Intensive Care Med.

[B28] Anonymous (2007). Gesetz über den Verkehr mit Arzneimitteln (Arzneimittelgesetz – AMG). BGBl.

[B29] Anonymous (2001). Directive 2001/20/EC of the European Parliament and of the Council of 4 April 2001 on the approximation of the laws, regulations and administrative provisions of the Member States relating to the implementation of good clinical practice in the conduct of clinical trials on medicinal products for human use. Off J Eur Communities.

[B30] Anonymous (2005). Commission Directive 2005/28/EC of 8 April 2005 laying down principles and detailed guidelines for good clinical practice as regards investigational medicinal products for human use, as well as the requirements for authorisation of the manufacturing or importation of such products. Off J Eur Union.

[B31] Vandenbroucke JP, von Elm E, Altman DG, Gotzsche PC, Mulrow CD, Pocock SJ, Poole C, Schlesselman JJ, Egger M (2007). Strengthening the Reporting of Observational Studies in Epidemiology (STROBE): explanation and elaboration. PLoS Med.

[B32] Iwanowski P, Budaj A, Czlonkowska A, Wasek W, Kozlowska-Boszko B, Oledzka U, Maselbas W (2008). Informed consent for clinical trials in acute coronary syndromes and stroke following the European Clinical Trials Directive: investigators' experiences and attitudes. Trials.

